# Deprivation, essential and non-essential activities and SARS-CoV-2 infection following the lifting of national public health restrictions in England and Wales

**DOI:** 10.3310/nihropenres.13445.1

**Published:** 2023-09-28

**Authors:** Susan Hoskins, Sarah Beale, Vincent Nguyen, Yamina Boukari, Alexei Yavlinsky, Jana Kovar, Thomas Byrne, Wing Lam Erica Fong, Cyril Geismar, Parth Patel, Anne M. Johnson, Robert W. Aldridge, Andrew Hayward

**Affiliations:** 1 Centre for Public Health Data Science, University College London, London, England, NW1 2DA, UK; 2 Institute of Epidemiology and Health Care, University College London, London, England, WC1E 7HB, UK; 3 Institute for Global Health, University College London, London, England, WC1N 1EH, UK

**Keywords:** SARS-CoV-2, Covid-19, deprivation, public transport, work, hospitality, leisure

## Abstract

**Background:**

Individuals living in deprived areas in England and Wales undertook essential activities more frequently and experienced higher rates of SARS-CoV-2 infection than less deprived communities during periods of restrictions aimed at controlling the Alpha (B.1.1.7) variant. We aimed to understand whether these deprivation-related differences changed once restrictions were lifted.

**Methods:**

Among 11,231 adult Virus Watch Community Cohort Study participants multivariable logistic regressions were used to estimate the relationships between deprivation and self-reported activities and deprivation and infection (self-reported lateral flow or PCR tests and linkage to National Testing data and Second Generation Surveillance System (SGSS)) between August – December 2021, following the lifting of national public health restrictions.

**Results:**

Those living in areas of greatest deprivation were more likely to undertake essential activities (leaving home for work (aOR 1.56 (1.33 – 1.83)), using public transport (aOR 1.33 (1.13 – 1.57)) but less likely to undertake non-essential activities (indoor hospitality (aOR 0.82 (0.70 – 0.96)), outdoor hospitality (aOR 0.56 (0.48 – 0.66)), indoor leisure (aOR 0.63 (0.54 – 0.74)), outdoor leisure (aOR 0.64 (0.46 – 0.88)), or visit a hairdresser (aOR 0.72 (0.61 – 0.85))). No statistical association was observed between deprivation and infection (P=0.5745), with those living in areas of greatest deprivation no more likely to become infected with SARS-CoV-2 (aOR 1.25 (0.87 – 1.79).

**Conclusion:**

The lack of association between deprivation and infection is likely due to the increased engagement in non-essential activities among the least deprived balancing the increased work-related exposure among the most deprived. The differences in activities highlight stark disparities in an individuals’ ability to choose how to limit infection exposure.

## Plain English summary

Individuals living in deprived areas of England and Wales left home to go to work and used public transport more frequently than people living in less deprived areas of the country when under tight lockdown restrictions. They were also more likely to develop SARS-CoV-2 infection. Understanding whether these differences changed once restrictions were lifted is important to understand whether deprivation-related discrepancies in infection risk changed throughout the pandemic. We found that, after the removal of lockdown restrictions, people living in areas of the greatest deprivation continued to leave home for work or use public transport more frequently than those not living in areas of deprivation but they were less likely to visit either indoor or outdoor hospitality or leisure venues such as cafes, restaurants, bars, cinemas, theatres or visit a hairdresser or beautician than people living in areas with little deprivation. They were no longer more likely than those living in areas with little deprivation to become infected with SARS-CoV-2. This is likely because people living in areas with little deprivation were visiting hospitality and leisure venues more frequently than during lockdown and were increasing their exposure to infection in these settings, balancing out the increased infection risk posed through work and public transport to those living in deprived areas. The fact that people living in areas of deprivation were most likely exposed to SARS-CoV-2 infection through essential activities like work and public transport use while people living in areas with little deprivation were most likely exposed to infection through non-essential activities such as visiting a restaurant, pub, cinema or theatre, highlights stark disparities in an individuals’ ability to choose how to limit infection exposure based on their deprivation status.

## Introduction

The first and second wave of the SARS-CoV-2 pandemic were largely spent under periods of national and regional ‘lockdown’ restrictions in the UK. Restrictions included advice to work from home where possible, travel restrictions, closure of non-essential businesses and leisure venues, restrictions on mixing socially and social distancing measures in public spaces ^1^. The aim of intense restrictions was to minimise transmission of SARS-CoV-2 and protect individuals from disease acquisition, morbidity and mortality, yet throughout the pandemic individuals living in areas of socioeconomic deprivation have experienced higher rates of infection and mortality than those in less deprived communities ^2– 23^.

Like seasonal respiratory infections, activities which increase social-mixing, such as working outside the home, visiting shops, using public transport or visiting hospitality and leisure venues increase the odds of acquisition of SARS-CoV-2 outside the home, likely through increased contact with infectious individuals and through aerosol transmission ^24– 27^. Socioeconomic deprivation, however, likely influences individuals’ ability to stay at home -for example, as a result of lower ability to work from home and greater reliance on public transport. During the first wave, communities living with higher social deprivation in the United States were less able to change their work settings and during the second wave (September 2020 – end April 2021) of the UK epidemic, individuals living in the lowest level of deprivation had to leave their home to undertake essential activities more frequently than less deprived communities: they had to leave their homes for work 1.2 times more frequently, use public transport up to six times more frequently, and go to essential shops 1.13 times more frequently ^28, 29^. Differential exposure to activities outside the home, is likely to have contributed to higher rates of infections, and consequently hospitalisations and deaths from COVID-19, in deprived communities during this time ^3– 5^. 

On 19 ^th^ July 2021, colloquially referred to as ‘Freedom Day’, non-pharmaceutical interventions (NPI’s) including the advice to work from home where possible, the closure of a range of non-essential businesses such as hospitality and leisure venues and restrictions on social gatherings were lifted in England. Wales lifted restrictions on 7 ^th^ August 2021. To our knowledge, no work has examined deprivation-related differences in activities or infections since the removal of all national restrictions which commenced in many countries in the middle of 2021.

By analysing whether deprivation was associated with non-household activities in the period following Freedom Day (1 ^st^ September – 16 ^th^ December of 2021), we aimed to understand whether the differences in activities undertaken outside the home which increase the odds of acquisition of SARS-CoV-2 during the period under national restrictions changed once restrictions were lifted. In addition, we sought to assess whether an increased odds of infection with SARS-COV-2 for individuals living in deprived areas was observed in the post Freedom Day period. 

## Methods

### Patient and public involvement

Due to constraints related to conducting research with a wide remit during the COVID-19 pandemic, patients and/or the public were not involved in the design or dissemination of this study.

### Study design and setting

The analyses are based on the Virus Watch Community Cohort in England and Wales, the detailed methodology of which, including eligibility criteria, recruitment and follow-up methods, is described elsewhere ^30^. Briefly, the study recruits whole households with detailed baseline information, weekly surveys of symptoms and self-reported positive SARS-COV-2 tests (PCR or lateral flow) conducted through the national tracing programme, linkage to the national testing data-set, and monthly questionnaires on social activity patterns during the preceding week. At the time of this study, Virus Watch had recruited 58, 628 adult and child individuals.

### Study population

***Inclusion criteria.*** Within the Virus Watch community cohort study, participants were included in the current study if they were aged 18 years and above and had completed three monthly behavioural surveys following the declaration of “Freedom Day” (completed during the periods 22/09/2021 – 29/09/2021, 19/10/2021 – 26/10/2021 and 16/11/2021 – 23/11/2021) and cases were included if testing PCR or lateral flow positive between 01/09/2021 and 16/12/2021 (before the wide-spread circulation of the Omicron variant).

***Exclusion criteria.*** Participants were excluded if there was evidence of recent infection in the previous three months (reported a positive PCR or lateral flow test in the 90 days before 01/09/2021) signally likely natural immunity. We did not include responses from the August survey as many participants were on holiday and survey completion rates were low.

### Exposure

The exposure of interest, deprivation, was derived using English or Welsh Index of Multiple Deprivation (IMD) quintiles 2019 ^31, 32^. The IMD combines official data on small local areas for seven dimensions of deprivation (i.e., income, employment, education, health, crime, barriers to housing and services, and the living environment). Overall scores across these dimensions are used to rank areas from the most deprived to the least deprived. Virus Watch participants’ postcodes were linked with the May 2020 ONS Postcode Lookup file ^33^ to derive IMD quintiles. Consequently, only participants who provided a valid postcode at the beginning of the study were included in our analyses. IMD were provided as quintiles in this analysis (1=most deprived, 5=least deprived).

### Outcomes

Survey respondents were requested to report the number of days on which they engaged in various activities during the week leading up to each monthly activity survey, including attending work outside their homes. Composite variables based on multiple items were created for the following activities: taking public transport (use of taxi, bus, over and underground rail or tram and air travel), use of shared car with a non-household member, indoor hospitality (eating in an indoor restaurant, café or canteen; going to an indoor bar, pub or club; and going to an indoor party), outdoor hospitality (eating in an outdoor restaurant, café, or canteen; going to an outdoor bar, pub or club; and going to an outdoor party), indoor leisure (attending a gym, the theatre, the cinema, a concert or sports event), outdoor leisure (outdoor team sport), non-social activities (visiting a barber, hairdresser, beautician or nail salon). 

To estimate overall activity patterns during the period immediately after Freedom Day, the weekly frequency of each activity was calculated by averaging relevant data from the three surveys. The following binary outcomes were then classified based on the frequency distribution for each composite activity variable: leaving home to go to work or education (no, any), using public or shared transport (none, any), visiting indoor hospitality settings (up to once a week, more than once a week), outdoor hospitality settings (none, any), indoor leisure settings (none, any), undertaking outdoor leisure activities (none, any), and visiting non-social settings (none, any) in the week prior to the survey. 

To examine the association between deprivation and infection, SARS-CoV-2 infection status was binary coded (yes/no evidence of infection) based on any of the following: i) a positive self-reported PCR test, ii) a positive self-reported lateral flow test, iii) a positive PCR or lateral flow test from data linkage to the Second Generation Surveillance System (SGSS), which contains official data regarding SARS-CoV-2 test results from hospitalisations (Pillar 1) and national community testing (pillar 2). Linkage was conducted by NHS Digital.

### Statistical methods

We used logistic regressions models to examine the association between deprivation and undertaking non-household activities and between deprivation and infection. For both sets of analyses, the least deprived quintile (IMD 5) was used as the reference category for level of deprivation.

***Deprivation and activities.*** We undertook separate logistic regression models for each of the activities (leaving home to go to work, car sharing and each of the composite activities). Univariable analyses was performed to examine the relationship between deprivation and the proportion undertaking each activity on a weekly basis. Multivariable models were adjusted for variables relevant to each activity. Where included, age was classified as adult of working age (18–64 years) or adult of retired age (65 years and above). Region was derived from linking participants’ postcode to ONS national region using the May 2020 National Statistics Postcode Lookup file (14).

The model examining the association between deprivation and leaving home for work was adjusted by sex, region, living with children, and area of residence, with a model additionally examining the effect of age. Sex was considered a relevant a priori potential confounder. Geographic region and area of residence (rural, urban, or conurbation) were controlled for as the local prevalence of infection likely determines the risk associated with doing any activities (separate to the actual risk of the activity itself). We controlled for the presence of children (<18 years) in the household due to the likely influence of COVID-19-related school closures on the working patterns of parents and carers. The additional model adjusting for the effect of age was included due to a plausible relationship between both IMD and working status.

We adjusted the use of the public transport and car share models by sex, region and area to take account of likely differential use of public transport in different geographical regions and differences between rural, urban and conurban areas. We conducted a further model additionally adjusted for age and employment status as, although having to leave home for employment is likely on the causal pathway between deprivation and use of public transport or car sharing, employment status is likely also related to both deprivation status and public transport use.

All other activity models (indoor hospitality use, outdoor hospitality use, indoor leisure, outdoor leisure and non-social activities) were each adjusted for age, on the basis that age is significantly related to activity levels, sex a priori, and living with children and living alone under the hypothesis that living with others is likely to affect the ability to undertake other activities (either reducing the opportunities to go out if looking after children or increasing the need to seek social engagement outside the home if living alone). 

Missing data were sparse and for ease of comparisons to the adjusted activity models participants with missing data were not included in the univariate analysis of the association between deprivation and activity, nor in the multivariate adjusted models.

***Deprivation and infection.*** For the infection analysis, we undertook univariable analyses comparing the proportion with evidence of infection according to deprivation status. We used multivariable logistic regression to adjust for age, sex and vaccination status, a priori, region due to the likely influence of regional variations in prevalence rates affecting infection risk, area of residence, living alone or living with children due to known associations with both deprivation and infection. Missing data were sparse and while shown in the univariate analyses and for each co-variable, participants with missing data were not included in the multivariate adjusted model.

All analyses were carried out using STATA version 16. 

### Ethical considerations

This study has been approved by the Hampstead NHS Health Research Authority Ethics Committee, Ethics approval number—20/HRA/2320. Written consent or assent was obtained at study registration for all aspects of the study. Participants were informed when providing consent that their de-identified data would be processed by the study team within a secure research environment for research purposes and overall study results without identifiable information would be published as scientific articles and presentations at scientific meetings.

## Results

Table 1 shows the characteristics of study participants (n=11,231). The cohort was made up of 57% females and 53% of study participants were of retired age. Participants came largely from the East of England (23%), the South East (19%), the North West (11%) and London (11%). Just under half of participants (46%) lived in an urban area. The majority (75%) of participants lived with someone and few (6%) lived with children. Nearly all (94%) participants had received at least once vaccine dose at entry to this study period and the majority (60%) were in employment. More than two-thirds of participants lived in areas with some level of deprivation, with 8% living in areas classified as within the most deprived quintile. 

**Table 1.** Characteristics of participants.

Characteristic	Category	Number in cohort (%) N=11,231
Deprivation score (IMD quintile) 1= most deprived	1 2 3 4 5	851 (8%) 1,589 (14%) 2,290 (20%) 3,032 (27%) 3,469 (31%)
Age (years)	18 – 64 years 65 and above	5,293 (47%) 5,938 (53%)
Sex	Male Female Missing	4,800 (43%) 6,390 (57%) 41
Region	East Midlands East of England London North East North West South East South West Wales West Midlands Yorkshire and The Humber	1,065 (9%) 2,551 (23%) 1,201 (11%) 581 (5%) 1,192 (11%) 2,210 (19%) 924 (8%) 266 (2%) 626 (6%) 615 (5%)
Area of residence	Rural Urban Conurbation	2,886 (26%) 5,209 (46%) 3,136 (28%)
Living with children	No Yes	10,505 (94%) 726 (6%)
Lives alone	No Yes	8,413 (75%) 2,818 (25%)
Vaccine status	No Yes	659 (6%) 10,572 (94%)
Any employment	No Yes	6,768 (60%) 4,463 (40%)

Table 2.1 – Table 2.8 Univariate and adjusted relationships between deprivation and non-household activities

**Table 2.1.** Leaving home for work, univariable and multivariable association with deprivation.

Characteristic	Category	Number in cohort N=11,190	Number (%) who ever leave home for work n=4,037	Unadjusted OR, 95% CI, p	OR adjusted for sex, region, living with children, area 95% CI, p value	Sensitivity model OR adjusted for age, sex, region, living with children, area 95% CI, p value
Deprivation score (IMD quintile) 1= most deprived	1 2 3 4 5	846 (8%) 1,582 (14%) 2,279 (20%) 3,020 (27%) 3,463 (31%)	365 (43%) 645 (41%) 850 (37%) 1,060 (35%) 1,117 (32%)	1.59 (1.37 – 1.86) 1.45 (1.28 – 1.64) 1.25 (1.12 – 1.39) 1.14 (1.02 – 1.26) 1.00 P<0.0001	1.56 (1.33 – 1.83) 1.36 (1.20 – 1.55) 1.25 (1.11 – 1.39) 1.13 (1.02 – 1.26) 1.00 P<0.0001	1.26 (1.06 – 1.49) 1.16 (1.01 – 1.34) 1.14 (1.01 – 1.29) 1.06 (0.95 – 1.19) 1.00 P=0.0402

**Table 2.2.** Any public transport use in a week, univariable and multivariable association with deprivation.

Characteristic	Category	Number in cohort N=11,190	Number (%) using any public transport/week n=5,405	Unadjusted OR, 95% CI, p	OR adjusted Sex, region, area 95% CI, p value	Sensitivity OR adjusted for Sex, region, area, employment status, age 95% CI, p value
Deprivation score (IMD quintile) 1= most deprived	1 2 3 4 5 Missing	846 (8%) 1,582 (14%) 2,279 (20%) 3,020 (27%) 3,463 (31%)	499 (59%) 824 (52%) 1,092 (48%) 1,408 (47%) 1,582 (47%)	1.71 (1.47 – 1.99) 1.29 (1.15 – 1.46) 1.09 (0.98 – 1.22) 1.04 (0.94 – 1.15) 1.00 P<0.0001	1.33 (1.13 – 1.57) 1.01 (0.89 – 1.15) 1.05 (0.94 – 1.17) 1.01 (0.91 – 1.11) 1.00 P=0.0100	1.29 (1.10 – 1.52) 0.99 (0.87 – 1.13) 1.03 (0.92 – 1.16) 1.00 (0.90 – 1.11) 1.00 P=0.0240

**Table 2.3.** Any car sharing with someone outside of your household, univariable and multivariable association with deprivation.

Characteristic	Category	Number in cohort N=11,190	Number (%) who ever car share n=6,638	Unadjusted OR, 95% CI, p	OR adjusted Sex, region, area 95% CI, p value	Sensitivity OR adjusted for Sex, region, area, employment status, age 95% CI, p value
Deprivation score (IMD quintile) 1= most deprived	1 2 3 4 5 Missing	846 (8%) 1,582 (14%) 2,279 (20%) 3,020 (27%) 3,463 (31%)	472 (56%) 866 (55%) 1,395 (61%) 1,797 (59%) 2,108 (61%)	0.81 (0.69 – 0.94) 0.78 (0.69 – 0.88) 1.01 (0.91 – 1.13) 0.94 (0.85 – 1.04) 1.00 P=0.0001	0.82 (0.70 – 0.96) 0.81 (0.71 – 0.91) 1.05 (0.94 – 1.17) 0.96 (0.86 – 1.06) 1.00 P=0.0003	0.83 (0.71 – 0.97) 0.81 (0.72 – 0.92) 1.05 (0.94 – 1.18) 0.96 (0.87 – 1.06) 1.00 P=0.0006

**Table 2.4.** Visiting indoor hospitality more than once a week, univariable and multivariable association with deprivation.

Characteristic	Category	Number in cohort N=11,190	Number (%) visiting indoor hospitality > 1/week, n=4,801	Unadjusted OR, 95% CI, p	OR adjusted for age, sex, living with children, living alone, area 95% CI, p value
Deprivation score (IMD quintile) 1= most deprived	1 2 3 4 5 Missing	846 (8%) 1,582 (14%) 2,279 (20%) 3,020 (27%) 3,463 (31%)	359 (42%) 630 (39%) 951 (42%) 1,301 (43%) 1,560 (45%)	0.89 (0.77 – 1.05) 0.81 (0.72 – 0.91) 0.87 (0.79 – 0.97) 0.92 (0.84 – 1.02) 1.00 P=0.0072	0.82 (0.70 – 0.96) 0.76 (0.67 – 0.86) 0.86 (0.77 – 0.96) 0.91 (0.83 – 1.01) 1.00 P=0.0002

**Table 2.5.** Any outdoor hospitality visited in a week, univariable and multivariable association with deprivation.

Characteristic	Category	Number in cohort N=11,190	Number (%) visiting any outdoor hospitality/ week n=4,558	Unadjusted OR, 95% CI, p	OR adjusted for age, sex, living with children, living alone, area 95% CI, p value
Deprivation score (IMD quintile) 1= most deprived	1 2 3 4 5 Missing	846 (8%) 1,582 (14%) 2,279 (20%) 3,020 (27%) 3,463 (31%)	272 (32%) 581 (37%) 955 (42%) 1,236 (41%) 1,514 (44%)	0.61 (0.52 – 0.71) 0.75 (0.66 – 0.84) 0.93 (0.83 – 1.03) 0.89 (0.81 – 0.98) 1.00 P<0.0001	0.56 (0.48 – 0.66) 0.71 (0.63 – 0.80) 0.91 (0.82 – 1.02) 0.88 (0.79 – 0.97) 1.00 P<0.0001

**Table 2.6.** Any indoor leisure activities in a week, univariable and multivariable association with deprivation.

Characteristic	Category	Number in cohort N=11,190	Number (%) undertaking any indoor leisure/ week n=4,636	Unadjusted OR, 95% CI, p	OR adjusted for age, sex, living with children, living alone, area 95% CI, p value
Deprivation score (IMD quintile) 1= most deprived	1 2 3 4 5 Missing	846 (8%) 1,582 (14%) 2,279 (20%) 3,020 (27%) 3,463 (31%)	318 (38%) 576 (36%) 941 (41%) 1,249 (41%) 1,552 (45%)	0.74 (0.64 – 0.87) 0.71 (0.62 – 0.79) 0.87 (0.78 – 0.96) 0.87 (0.79 – 0.96) 1.00 P<0.0001	0.63 (0.54 – 0.74) 0.63 (0.56 – 0.72) 0.85 (0.76 – 0.95) 0.85 (0.77 – 0.94) 1.00 P<0.0001

**Table 2.7.** Any outdoor leisure activities in a week, univariable and multivariable association with deprivation.

Characteristic	Category	Number in cohort N=11,190	Number (%) undertaking any outdoor leisure/ week n=824	Unadjusted OR, 95% CI, p	OR adjusted for age, sex, living with children, living alone, area 95% CI, p value
Deprivation score (IMD quintile) 1= most deprived	1 2 3 4 5 Missing	846 (8%) 1,582 (14%) 2,279 (20%) 3,020 (27%) 3,463 (31%)	46 (5%) 89 (6%) 153 (7%) 245 (8%) 291 (8%)	0.63 (0.45 – 0.86) 0.65 (0.51 – 0.83) 0.78 (0.64 – 0.96) 0.96 (0.81 – 1.15) 1.00 P=0.0002	0.64 (0.46 – 0.88) 0.65 (0.51 – 0.83) 0.78 (0.64 – 0.96) 0.96 (0.81 – 1.15) 1.00 P=0.0004

**Table 2.8.** Any non-social non-household activity in a week, univariable and multivariable association with deprivation.

Characteristic	Category	Number in cohort N=11,190	Number (%) visiting any non-social venue/week n=3,862	Unadjusted OR, 95% CI, p	OR adjusted for age, sex, living with children, living alone, area 95% CI, p value
Deprivation score (IMD quintile) 1= most deprived	1 2 3 4 5 Missing	846 (8%) 1,582 (14%) 2,279 (20%) 3,020 (27%) 3,463 (31%)	263 (31%) 484 (31%) 782 (34%) 1,032 (34%) 1,301 (38%)	0.75 (0.64 – 0.88) 0.73 (0.65 – 0.83) 0.87 (0.78 – 0.97) 0.86 (0.78 – 0.96) 1.00 P<0.0001	0.72 (0.61 – 0.85) 0.72 (0.63 – 0.82) 0.85 (0.76 – 0.96) 0.85 (0.77 – 0.95) 1.00 P<0.0001

### Deprivation and activities ( Figure 1)

There was strong evidence that living in areas with any level of deprivation was associated with having to leave home to go to work, the odds increasing with each level of deprivation, the greatest found in those who are most deprived (OR 1.59 (1.37 – 1.86) ( Table 2.1). This association remained strong for all levels of deprivation after multivariable adjustments (aOR 1.56 (1.33 – 1.83) for the most deprived). The strength of the increase in odds was reduced across all strata (aOR 1.26 (1.06 – 1.49) for most deprived) when additionally adjusting for age but remained significant.

**Figure 1.**
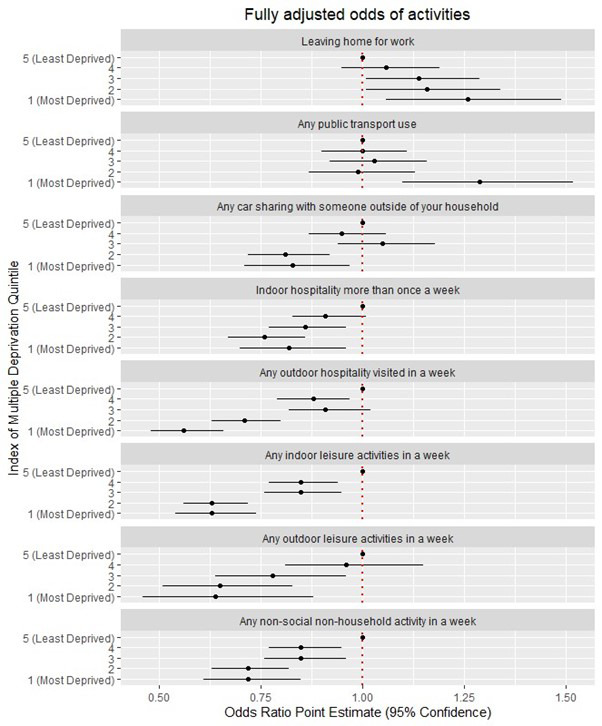
Fully adjusted relationship between deprivation and activities (adjustments as per Table 2.1– Table 2.8).

There was strong evidence that deprivation was independently associated with public transport use, the effect being particularly strong among those living with the greatest level of deprivation (OR 1.71 (1.47 – 1.99)) ( Table 2.2). After controlling for the effects of sex, region and area, the size of the effect reduced (aOR 1.33 (1.13 – 1.57) but remained significant. There was little change after additionally controlling for age and employment status. Correspondingly, those living with the greatest levels of deprivation were less likely to report car-sharing with a non-household member than the least deprived (OR 0.81 (0.69 – 0.94) ( Table 2.3). The size and strength of the effect remained largely unchanged (aOR 0.83 (0.71 – 0.97) after adjusting for sex, region, area, employment status and age.

Those living across nearly all strata of deprivation reported undertaking fewer hospitality, leisure or non-social activities compared to the least deprived, with decreasing odds of undertaking activities with increasing levels of deprivation ( Table 2.4 – Table 2.8). The effect sizes and strength of associations were minimally altered after multivariable adjustments, with the following reduced odds observed for the most deprived compared to the least deprived: indoor hospitality (aOR 0.82 (0.70 – 0.96)), outdoor hospitality (aOR 0.56 (0.48 – 0.66)), indoor leisure (aOR 0.63 (0.54 – 0.74)), outdoor leisure (aOR 0.64 (0.46 – 0.88)), non-social activities (aOR 0.72 (0.61 – 0.85)) ( Table 2.4 – Table 2.8).

### Deprivation and infection

During the period (1 ^st^ September – 16 ^th^ December 2021), among 11,231 participants, 482 cases were identified ( Table 3). Although an increase in odds of infection was observed across all strata of deprivation (OR 1.36 (0.96 – 1.92) among the most deprived), the observed association for deprivation was non-significant in this time-period. After adjusting for all potential confounders, those living in the greatest level of deprivation appeared to continue to have an increased odds of infection but the results remained non-significant (aOR 1.25 (0.87 – 1.79) ( Table 3). 

**Table 3.** Association between deprivation and infection (adjustments: age, sex, region, area of residence, vaccination status, living: with children; alone).

Characteristic	Category	Number in cohort (%) N=11,231	Number of cases n=482 (%)	Unadjusted OR, 95% CI, p	Adjusted OR, 95% CI, p value, n=11,190
Deprivation score (IMD quintile) 1= most deprived	1 2 3 4 5	851 (8%) 1,589 (14%) 2,290 (20%) 3,032 (27%) 3,469 (31%)	44 (5%) 69 (4%) 94 (4%) 141 (5%) 134 (4%)	1.36 (0.96 – 1.92) 1.13 (0.84 – 1.52) 1.07 (0.82 – 1.39) 1.21 (0.95 – 1.55) 1.00 P=0.3740	1.25 (0.87 – 1.79) 1.06 (0.78 – 1.44) 1.11 (0.84 – 1.47) 1.20 (0.94 – 1.54) 1.00 P=0.5745
Age (years)	18 – 64 years 65 and above	5,293 (47%) 5,938 (53%)	324 (6%) 158 (3%)	2.38 (1.96 – 2.89) 1.00 P<0.0001	1.99 (1.62 – 2.45) 1.00 P<0.0001
Sex	Male Female Missing	4,800 (43%) 6,390 (57%) 41	206 (4%) 275 (4%)	1.00 1.00 (0.83 – 1.21) P=0.9754	1.00 1.01 (0.83 – 1.22) P=0.9446
Region	East Midlands East of England London North East North West South East South West Wales West Midlands Yorkshire and The Humber	1,065 (9%) 2,551 (23%) 1,201 (11%) 581 (5%) 1,192 (11%) 2,210 (19%) 924 (8%) 266 (2%) 626 (6%) 615 (5%)	35 (3%) 92 (4%) 57 (5%) 35 (6%) 62 (5%) 102 (5%) 33 (4%) 18 (7%) 33 (5%) 15 (2%)	1.00 1.10 (0.74 – 1.64) 1.47 (0.95 – 2.25) 1.87 (1.17 – 3.05) 1.61 (1.06 – 2.46) 1.42 (0.96 – 2.11) 1.09 (0.67 – 1.77) 2.14 (1.19 – 3.83) 1.64 (1.01 – 2.66) 0.74 (0.39 – 1.36) P=0.0028	1.00 1.13 (0.76 – 1.69) 1.13 (0.69 – 1.81) 1.87 (1.15 – 3.05) 1.59 (1.04 – 2.47) 1.57 (1.05 – 2.33) 1.23 (0.75 – 2.02) 2.52 (1.39 – 4.57) 1.54 (0.94 – 2.52) 0.71 (0.38 – 1.32) P=0.0014
Living with children	No Yes	10,505 (94%) 726 (6%) 4	393 (4%) 89 (12%)	1.00 3.59 (2.82 – 4.59) P<0.0001	1.00 2.34 (1.79 – 3.05) P<0.0001
Lives alone	No Yes	8,413 (75%) 2,818 (25%)	402 (5%) 80 (3%)	1.72 (1.35 – 2.19) 1.00 P<0.0001	1.57 (1.22 – 2.03) 1.00 P=0.0003
Vaccine status	No Yes	659 (6%) 10,572 (94%)	27 (4%) 455 (4%)	0.95 (0.64 – 1.41) 1.00 P=0.7981	0.91 (0.61 – 1.37) 1.00 P=0.6580
Area of residence	Rural Urban Conurbation	2,886 (26%) 5,209 (46%) 3,136 (28%)	106 (4%) 216 (4%) 160 (5%)	1.00 1.13 (0.89 – 1.44) 1.41 (1.09 – 1.81) P=0.0199	1.00 1.07 (0.83 – 1.36) 1.37 (1.01 – 1.86) P=0.1100
Any employment	No Yes	6,768 (60%) 4,463 (40%)	223 (3%) 259 (6%)	1.00 1.81 (1.51 – 2.17) P<0.0001	-

## Discussion

We observed stark differences in the types of essential and non-essential activities undertaken by those living in deprived areas and those living in areas of little deprivation following the lifting of restriction on Freedom Day. We did not find an association between deprivation and infection in the three-month period following Freedom Day. The lack of observed association between deprivation and infection is different to that observed earlier in the pandemic and is likely related to the clear differences in behaviour we observed once restrictions were lifted ^29^. Those living in areas with the greatest level of deprivation, as was observed in the first two waves of the UK pandemic, continued to undertake activities known to increase risk of acquisition of SARS-CoV-2 (including leaving home for work and using public transport) ^29^. But post Freedom Day, people living in areas with less deprivation engaged in more non-work non-transport activities associated with increased risk of SARS-CoV-2 infection than those most deprived (undertaking indoor hospitality and indoor leisure activities). It is likely that this increased engagement in social activities among those living in least deprived areas, balances the increased risk of work-related exposure in those living in more deprived areas such that the risk of infection with SARS-CoV-2 becomes more evenly spread making deprivation less of an infection risk.

Our findings are similar to others which demonstrate differing activity changes by deprivation during the pandemic. In El Paso, Texas, throughout the pandemic, recreational walking and use of green spaces was more greatly reduced in neighbourhoods with more deprivation than in less deprived neighbourhoods ^34^. In Seoul, South Korea, the frequency of subway use during the pandemic decreased only in the least deprived areas, suggesting a disparity in the ability to socially-distance by deprivation, similar to our own findings ^35^. Our study, found stark disparities by deprivation status in activities undertaken such as working outside the home, use of public transport, and frequency of hospitality and leisure activities. This unequal impact through human mobility according to socio-economic status, affecting the ability to choose whether to socially distance or not, has been called the “luxury nature” of social-distancing ^36^. 

### Strengths and limitations

Our exposure measure, deprivation, was measured during baseline surveys and our outcomes (either activities or infection) were measured during the post-Freedom Day period. It is possible that participants deprivation status changed throughout the pandemic due to residential moving but we did not capture updated deprivation area data. Activities and behaviours are self-reported and therefore subject to recall bias and social desirability bias although data examined during the first wave of the pandemic in Germany were found to support the use of self-reported contact survey data to reflect infection dynamics ^37^. We tried to minimise recall bias by asking about activities in the previous seven days. The activities were sampled at three points during the period post Freedom Day but did not include survey results from the month of August, a holiday period in the UK, as response rates were low. We may have missed any immediate increase in activities around Freedom Day. Moreover, data taken from one-week time-periods in a monthly survey may not be representative of activities throughout a month. We sought to minimise this bias by taking an average of activities engaged in across the three months. Using self-reported and linked data on test results from the national testing system allowed better ascertainment of infections than cohort data alone and increased ascertainment of infection data supports increasingly accurate assessment of the importance of deprivation. However, the period of follow-up for infections between Freedom Day and before the onset of the more infectious Omicron variant was short so we may have been underpowered to examine an association between deprivation and infection in the post-Freedom Day period. Virus Watch survey respondents were not demographically representative of the population, with a lower proportion of the survey samples drawn from the most deprived communities (8%) compared with the least deprived (30%), potentially affecting generalisability of responses from those living in the most deprived areas as well as statistical power. Finally, Virus Watch is a voluntary cohort likely subject to a degree of self-selection bias and it is possible that our cohort may include individuals with a higher socioeconomic status living within the more deprived areas and we may be underestimating the true effect size. Future studies that are able to capture time-updated information on deprivation status and examine infections over a longer period of time, or throughout the Omicron wave when prevalence and case numbers were higher will be of value to add power to this work and to refine this research further.

## Conclusion

We did not observe an associated increased odds of infection among those most deprived in the post-Freedom Day period, but the differences in activities undertaken highlight stark disparities in an individuals’ ability to choose how to limit exposure to infection. People living in most deprived settings undertook more essential infection-associated activities (leaving home for work and using public transport) while those living in the least level of deprivation undertook more infection-associated non-essential activities (going to an indoor or outdoor bar, pub, club, eating at an indoor restaurant, café or canteen, attending an indoor party, going to the cinema, a concert, the theatre or sports event or gym). Deprivation-related differences in exposure to SARS-CoV-2 via essential or non-essential activities likely reflect factors that constrain individual choice, such as car ownership, ability to work from home and disposable income. Measures to mitigate infection risk during essential activities are indicated to address deprivation-related inequalities during pandemics.

## Data Availability

We aim to share aggregate data in the form of findings via a “Findings so far” section on our website. We are sharing individual record-level data on the Office of National Statistics Secure Research Service, and given the sensitive content in our dataset for this study, we cannot release the data at the individual level. Access to use of the data whilst research is being conducted will be managed by the Chief Investigators (ACH and RWA) in accordance with the principles set out in the UK Research and Innovation Guidance on best practice in the management of research data. Data access requests can also be made directly to the Virus Watch chief investigators (ACH or RWA) at the following email address: viruswatch@ucl.ac.uk. The data along with the analysis code used will be provided to approved researchers. The dataset can be found here https://doi.org/10.57906/s5f5-nq13
