# Supplementary material for: Deprivation, essential and non-essential activities and SARS-CoV-2 infection following the lifting of national public health restrictions in England and Wales
Source: NIHR Open Res. 2023 Sep 28;3:46. [Version 1] doi: 10.3310/nihropenres.13445.1 (PMC10663878; doi:10.3310/nihropenres.13445.1)
Supplement: Peer review history

# Reviewer report: Reviewer response for version 1

**DOI:** 10.3310/nihropenres.14583.r30596

**Published:** 2023-11-08

**Authors:** Benjamin Wachtler, Florian Beese

**Affiliations:** 1 Department of Epidemiology and Health Monitoring, Robert Koch Institute, Berlin, Germany

This study reports the results of an analysis of deprivation-related differences in essential and non-essential activities after the lifting of restrictions of the individuals’ mobility during the COVID-19 pandemic in England and Wales. The second aim of this study was to assess the association between area-level socioeconomic deprivation and individual SARS-CoV-2 infection risk. The underlying data were derived from the Virus Watch Cohort, which includes monthly surveys and self-reported information on SARS-COV-2 infections. The results showed that those living in areas with higher socioeconomic deprivation had higher odds for essential activities after the restriction were lifted and those living in areas with lower levels of deprivation had higher odds for non-essential activities. In conclusion, this is a well conducted study that aimed to answer interesting research questions that are timely and relevant to public health. It uses high quality data and appropriate methods. Overall, the article is well-written and describes the methods used for generating the results in sufficient detail to evaluate the results.

 However, there are some minor issues and inconsistencies that might be amended to further improve the article.

**Overall:**

- The IMD primarily includes dimensions that are linked to socioeconomics and the authors also link the concepts of socioeconomic status and deprivation in the discussion. The authors might consider to consistently rephrase "deprivation" into "socioeconomic deprivation"

**Abstract:**

- The abbreviation "SGSS" is not necessary here, as it only appears once in the abstract.
- The last sentence in the plain English summary is very long. The authors might consider to rephrase it.

**Introduction:**

- The second paragraph seems like something is probably mixed up here. The authors state that higher deprived people in the US were less able to protect themselves and in the same sentence the authors state that people in the UK living in the lowest deprived areas had to leave their homes more often compared to less deprived. Presumably, the author meant that the individuals living in highly deprived areas had to leave their homes more frequently?

**Methods:**

- The authors state "The exposure of interest, deprivation, was derived using English or Welsh Index of Multiple Deprivation". We were wondering if there is any difference between those and if yes, what does that mean for the interpretation of the results?
- Why did the authors decide to categorise "visiting indoor hospitality settings" in "up to once a week" and "more than once a week" while the other items were categorised in "none" and "any"? It would be interesting to see some sensitivity analyses here with different categorisations and how results may change.
- The abbreviation "SGSS" is not necessary as it only appears once in the manuscript.
- "Univariable analyses was performed" in the section *Deprivation and activities* should be written "Univariable analyses were performed".
- Study population and sample population: It would be very helpful for the reader if the authors might consider to integrate a flow chart that describes the selection process that led to the sample population.
- Statistical analyses: We were wondering how the confidence intervals were estimated? How did the authors deal with design effects here particularly with household clustering? Were there any forms of weighting procedures in place? If the analyses were conducting by using the survey functionality of STATA, this should be stated here as well.
- Deprivation and infection: We were wondering why the multivariate analyses were adjusted for vaccination status? Vaccination status is known to be lower in regions with higher socioeconomic deprivation and might lead to a certain level of protection against infection, hence vaccination status might be on the causal pathway between deprivation and infection and perhaps should be regarded as mediator. Adjusting for vaccination status might lead to an over-adjustment and therefore an underestimation of the association.

**Results:**

- The authors state in the text that 60% of the participants were employed. However, this is inconsistent with the information given in Table 1, where only 40% were employed.
- The statement "More than two-thirds of participants lived in areas with some level of deprivation, with 8% living in areas classified as within the most deprived quintile." is unclear to us. What does "at least some level of deprivation" mean?
- The authors might consider to rephrase "Vaccine status" in "At least one vaccine dose" for clarification in table 1.
- Figure 1:
  - The authors might consider removing the plot title that is generated with ggplot2 as there is a figure caption below the figure. Furthermore, this plot title is not fully correct as the authors report odds ratios instead of odds. In addition, figures should be interpretable without reference to other sources. Please consider to state the exact adjustment sets for each model in the figure caption. The authors might as well consider to show both, the unadjusted and fully adjusted estimates here by using colour coding.
  - When plotting ratio estimates (which are scaled from 0 to infinity), the x-axis should be formatted as a log-scale. The reason is, that an odds ratio of 0.5 means half the odds and an odds ratio of 2 means doubled odds for the outcome. A log-scale would consider this and would equalize the distances between 0.5 and 0, and 0 and 2 respectively (equidistance). Using a normal scale when reporting ratio measures might result in a misleading graphical presentation of the results.
- The authors might consider to only refer to figure 1 when they report results of it in the text. It was confusing, that figure 1 was referred to in the subsection header while the result of the fully adjusted model (Sensitivity model) was reported at the end of the first paragraph in *Deprivation and activities (Figure 1).*
- Tables 2.1 to 2.8:
  - The authors should remove "Missing" from the category column as they do not report missing values.
  - The authors might think about to remove the first column and rename the second column "Deprivation" instead of "Category".
- It might be noteworthy that the Quintile 4 has nearly the same odds for infection than Quintile 1. The authors might consider reporting this finding and discuss it in the discussion section.

**Discussion:**

- The age distribution should be further discussed in the discussion section. For their analyses, the authors decided to categorise age in 18-64 and >=65, which is a very broad categorisation. It might make a difference here if the individual is in her or his twenties or just before the retirement age. How do the results change, when choosing another categorisation with consideration of different life stages or age as a continuous variable? The authors might consider to implement at least some lines in the discussion to address the possible role of age in their analyses.
- The authors used logistic regression models to estimate the association between essential and non-essential activities with deprivation. However, the prevalence estimates of the outcomes are high and thus, this might result in overestimated associations. The authors might consider to address this issue in the interpretation of their results.

Is the work clearly and accurately presented and does it cite the current literature?

Yes

If applicable, is the statistical analysis and its interpretation appropriate?

Yes

Are all the source data underlying the results available to ensure full reproducibility?

Yes

Is the study design appropriate and is the work technically sound?

Yes

Are the conclusions drawn adequately supported by the results?

Yes

Are sufficient details of methods and analysis provided to allow replication by others?

Yes

Reviewer Expertise:

Social epidemiology, health inequality research, public health

We confirm that we have read this submission and believe that we have an appropriate level of expertise to confirm that it is of an acceptable scientific standard, however we have significant reservations, as outlined above.

# Reviewer report: Reviewer response for version 1

**DOI:** 10.3310/nihropenres.14583.r30594

**Published:** 2023-10-30

**Authors:** Gerry McCartney

**Affiliations:** 1 College of Social Sciences, University of Glasgow, Glasgow, Scotland, UK

This a well conducted and very interesting study which compares the activities undertaken by people living in contrasting areas, as ranked by deprivation, during the acute phase of the pandemic. The finding of very different reasons for movement and coming into contact with others is important in explaining differential population exposures and inequalities in outcomes. 

 I have some comments below which I hope may be helpful: 

1. In the abstract and results it is reported that: "No statistical association was observed between deprivation and infection (P=0.5745), with those living in areas of greatest deprivation no more likely to become infected with SARS-CoV-2 (aOR 1.25 (0.87 – 1.79)." I think this somewhat confuses statistical precision with an important effect size. The best estimate of effect here is a 25% difference, but this is very imprecise and uncertain. I don't think it is accurate to say that there is no association. for example, if there was a bigger sample the conclusion might have been a 25% difference. I would urge the authors to consider reframing this as there being an uncertain relationship rather than there being no association. This finding also makes it through to the conclusion, where I would urge a similar framing. 
2. Although I see the authors have pointed to other studies which provide details of the underlying study, I think it is important to summarise the sampling method and the likely sampling biases with this kind of study (both in the methods and then reflected in the discussion-limitations section). This kind of study is particularly prone to differential non-response, and if quota sampling is used to up-weight particular demographics this can obscure underlying biases as it is assumed that responders in a similar demographic are similar in the outcomes of interest to non-responders, something that has been shown not to be the case in national health surveys. The very skewed numbers in each deprivation fifth suggest that this may be an important problem in the underlying study. This deserves more discussion in the limitations section. 
3. I wasn't clear why the age variable was dichotomised rather than used as a continuous variable - it would be useful for this to be explained further. Some of the other variables included as confounders could be interpreted as such, or could be interpreted as over-adjustment. For example, people are more likely to live in deprived areas if they have children - does this over-adjust for the effects of deprivation? For analysis of travel and contact with others, I think this might be overadjustment. For analysis of infection, it could be appropriate as children may have been an infection vector (but even that might be overadjustment as it is part of the experience of living in deprived areas - it depends on how the research question is articulated). In general, a clearer description of the exact research questions, causal theory (e.g. using a Directed Acyclic Graph), and justification for the adjustment for putative confounders would be helpful. 
4. The authors at times choose to use the word 'disparities' rather than inequalities - I'm not clear what the rationale for this is - the differences are systemic, avoidable and unfair, and in my view should be termed inequalities. 
5. The authors use area deprivation as a means of ranking the population to identify inequalities. This is appropriate, but has the marked limitation of misclassifying large proportions of the population (i.e. most deprived individuals do not live in the most deprived areas). If the authors have access to individual measures of socioeconomic position, I would strongly urge them to use these to rank and calculate inequalities. If not, the limitations of area based measures should be discussed in more detail. 
6. It may be useful to make the point that the changing exposure also coincides with the availability of vaccine, which meant that the higher exposure of people in less deprived areas due to leisure and other activities was in the context of better protection from the worse effects of the infection. 
7. It may be useful to identify that not all causes of infection are likely to have been identified through laboratory testing, and this may have been differentially experienced across social groups. 

Overall, this is a very important and well-conducted study which answers important questions on how and why inequalities in infection and mortality occurred during the first waves of the Covid-19 pandemic.

Is the work clearly and accurately presented and does it cite the current literature?

Yes

If applicable, is the statistical analysis and its interpretation appropriate?

Yes

Are all the source data underlying the results available to ensure full reproducibility?

Yes

Is the study design appropriate and is the work technically sound?

Yes

Are the conclusions drawn adequately supported by the results?

Partly

Are sufficient details of methods and analysis provided to allow replication by others?

Yes

Reviewer Expertise:

Public health, epidemiology, heterodox economics

I confirm that I have read this submission and believe that I have an appropriate level of expertise to confirm that it is of an acceptable scientific standard, however I have significant reservations, as outlined above.

# Reviewer report: Reviewer response for version 1

**DOI:** 10.3310/nihropenres.14583.r30598

**Published:** 2023-10-27

**Authors:** Aldo Rosano

**Affiliations:** 1 National Institute for the Analysis of Public Policies, Rome, Italy

The manuscript is well structured, the objectives are clearly stated, the methods are appropriate, but conclusions are not fully consistent with the results. The aim of the manuscript is to whether these deprivation-related differences in SARS-CoV-2 infection and undertaking essential activities changed once restrictions were lifted.

 Methods: it would be interesting to test the relationship between deprivation and SARS infection after the “Freedom day” also considering, in the same model, the various activities reported (leaving home for work, public transport use, etc) in order “to investigate community incidence of COVID-19 in relation to population movement and behaviours”.

 When analysing the use of public transport and car sharing the employment status was considered as adjusting factor. As mentioned by the authors employment status is correlated with the IMD, that combines information from the seven domains, including “employment Deprivation“. This aspect should be better discussed. IMD could be also (or in alternative) used as a contextual factor (e.g. calculated at county level) by adopting a multilevel modelling.

 Conclusions are focused on the main finding of the study “the lack of association between deprivation and infection”. Actually, it is about a lack of a statistically significant association, but a higher risk among most deprived subjects is confirmed, (aOR=1.26 for most deprived ones), and increasingly with higher level of deprivation. The lack of statistical significance is likely due to an insufficient power of the study. Any further consideration should consider this aspect. The conclusions seem to interpret the results as if deprivation was not a risk factor for Sars infection at all.

 On the other hand, to test the hypothesis “the increased engagement in non-essential activities among the least deprived may balance the increased work-related exposure among the most deprived” the authors could compare the periods before and after the “freedom day” and this is apparently possible as the Virus Watch cohort started in June 2020.

Is the work clearly and accurately presented and does it cite the current literature?

Yes

If applicable, is the statistical analysis and its interpretation appropriate?

Partly

Are all the source data underlying the results available to ensure full reproducibility?

Yes

Is the study design appropriate and is the work technically sound?

Yes

Are the conclusions drawn adequately supported by the results?

Partly

Are sufficient details of methods and analysis provided to allow replication by others?

Yes

Reviewer Expertise:

Epidemiology

I confirm that I have read this submission and believe that I have an appropriate level of expertise to confirm that it is of an acceptable scientific standard, however I have significant reservations, as outlined above.

# Reviewer report: Reviewer response for version 1

**DOI:** 10.3310/nihropenres.14583.r30485

**Published:** 2023-10-16

**Authors:** Tyra Grove Krause

**Affiliations:** 1 Department of Epidemiology Research, Statens Serum Institut, Copenhagen, Denmark

**Review: Deprivation, essential and non-essential activities and SARS-CoV-2 infection following the lifting of national public health restrictions in England and Wales.**

 This is a well-conducted cohort study comparing the essential and non-essential activities and infection risk in people living in socio-economic deprived areas and non- deprived area after lifting restrictions from 1 ^st^ September to 16the December 2021 in England and Wales when the Alpha variant circulated. The study is relevant from a public health perspective as it illustrates the difficulties in reducing transmission in deprived areas during a pandemic, as the population is more likely to have essential activities. The paper is well written and the methods are sound.

 Below are some suggestions for improvements:

**Abstract**

- Background:
  - When deprivation is mentioned the first time include socio economical deprivation.
  - line 2: Use area instead of communities. Suggest to change “We aimed to understand” to "We explored"
- Conclusion: suggest "may be due to" instead of "is likely due to".

**Introduction: **

- Suggest to delete "disease acquisition" I think it is included in morbidity.
- Last paragraph is rather long and complicated could be reduced to something like this:
  In this study we explored whether non-household activities and odds of infection differed for individuals living in deprived areas vs. non deprived areas in the period following Freedom day (1 ^st^ September- 16 ^th^ December 2021).

**Methods:**

- It seems that a relatively high proportion of the cohort is unemployed. I was wondering if those who are unemployed and not attending an education are excluded from the “leaving home for work” analysis?

**Discussion: **

- First paragraph suggest to alter to "we did not find a statistical significant association between infections and deprivation…"
- Would it be possible to describe the participants of the cohort with respect to the representativeness of the background population according to some other variables that IMD. It seems that a rather high proportion is unemployed is this because the population is also younger.

Is the work clearly and accurately presented and does it cite the current literature?

Yes

If applicable, is the statistical analysis and its interpretation appropriate?

I cannot comment. A qualified statistician is required.

Are all the source data underlying the results available to ensure full reproducibility?

Yes

Is the study design appropriate and is the work technically sound?

Yes

Are the conclusions drawn adequately supported by the results?

Yes

Are sufficient details of methods and analysis provided to allow replication by others?

Yes

Reviewer Expertise:

Infectious disease epidemiology, respiratory infections, vaccine effectiveness and safety

I confirm that I have read this submission and believe that I have an appropriate level of expertise to confirm that it is of an acceptable scientific standard.
